# Rural children active trachoma risk factors and their interactions

**DOI:** 10.11604/pamj.2016.24.128.8790

**Published:** 2016-06-09

**Authors:** Essey Kebede Muluneh, Temesgen Zewotir, Zerihun Bekele

**Affiliations:** 1 Department of Statistics, Bahir Dar University, Bahir Dar, Ethiopia; 2 School of Mathematics, Statistics and Computer Science, University of Kwazulu Natal, South Africa; 3 Central Statistical Authority, Addis Ababa, Ethiopia

**Keywords:** Association, cluster sampling, cumulative logit, design effect, proportional odds

## Abstract

**Introduction:**

Trachoma is a serious public health problem in rural Ethiopia. The aim of this investigation was to provide in-depth statistical analysis of the risk factors associated with active trachoma among children of age 1-9 years of Kedida Gamela district, in Ethiopia.

**Methods:**

A community based cross-sectional survey of trachoma was conducted in six selected rural kebeles of Kedida Gamela district, in Ethiopia from June 10-25, 2014. A total of 377 children (ages 1-9 years) were included in the study using two stage cluster sampling. All children were examined for trachoma by nurse data collectors supervised by ophthalmic supervisors using the WHO simplified clinical grading system. Ordinal survey logistic regression model was used to identify risk factors. Data analysis was done using SAS version 9.3.

**Results:**

The best fit proportional odds model was identified to be the main effects and two-way and three-way interactios. Keeping cattle in the house was found to have a protective effect (OR=0.138, p-value=0.0003). The household wealth will have a more protective effect if the child attends school. Washing face with soap and water once a day has equivalent protective effect as washing face three-or-more times a day with water only.

**Conclusion:**

The 2-way and 3-way significant interactions effects unfolded some of the controversies derived from similar studies on trachoma risk factors. The findings would suggest integrated effort to address two or three factors simultaneously is more fruitful than any novel intervention targeted to address a single risk factor.

## Introduction

Trachoma is the leading infectious cause of blindness worldwide. It is caused by infection with *Chlamydia trachomatis* and is characterized by inflammatory changes in the conjunctiva in children with subsequent scarring, corneal opacity and blindness in adults [1, 2]. The World Health Organization established an alliance for the Global Elimination of Trachoma by the year 2020 (GET 2020). The main goal of the alliance was to ensure that trachoma will cease to be disease of the public health importance by the year 2020. The diagnosis of trachoma is made on clinical grounds. Laboratory testing is typically unavailable or unaffordable for clinical care in areas where trachoma is endemic. Different studies have shown that the highest incidence of trachoma is generally in the poorest countries with those which have no good sanitation, water facility, and relatively low levels of economic development [3, 4]. Globally, 1.2 billion people live in endemic areas, 40.6 million people are suffering from active trachoma, and 48.5% of the global burden of active trachoma is concentrated in five countries: Ethiopia, India, Nigeria, Sudan and Guinea. Overall, Africa is the most affected continent with 27.8 million (68.5% of the 40.6 million) cases of active trachoma [5]. The prevalence studies on active trachoma showed variations from country to country. For instance, in Nigeria (2009), sample size, n=639, 35.7% (95% CI 32.0-39.6) [6]; Tanzania (2006), n=12 415, 9.1%, (95% CI 8.0-10.2) [7]; South Sudan (2007), n=7418, 64.5% (95% CI, 41.7- 87.8%) [8] and Ethiopia (2007), n=26163, 40.14%(95% CI 39.55 -40.73) [9]. A number of descriptive and informative studies on trachoma were conducted in Ethiopia. These studies have greatly contributed to our understanding of many aspects of the trachoma problem and the magnitude of trachoma [3, 9–15]. They helped us to understand the complexity of not only the problem but also the need for sustained and coordinated interventions to effectively prevent and control eye problems related to trachoma infection. However, all of these studies focused more on descriptive statistical analyses. But advanced statistical analysis is useful to measure the intrinsic and explicit effects of the socio-economic, demographic and environmental factors on the risk of trachoma. This study is hence aimed to assess the associations between the potential risk factors and active trachoma for children aged 1-9 years in rural Ethiopia. The study employed ordinal survey logistic regression analysis for in-depth investigation of the joint effect of two or more risk factors.

## Methods

### The data

We sought to conduct a community based cross-sectional survey of trachoma in Kedida Gamela district of Kembata Tambaro Zone in central Ethiopia from June 10-25, 2014. Kedida Gamela district is about 306 kilometers south-west of Addis Ababa, the capital city of Ethiopia. According to the 2007 population and housing census of Ethiopia, Kedida Gamela district have had population size of 89 391 with 44 589 males and 44,802 females; of which about 95% were living in the rural areas. The total number of children aged 1-9 years residing in rural areas of this district was approximately 10 886. The Kedida Gamela district was known for trachoma endemicity [15]. The study was undertaken among children aged 1-9 years old. Structured questionnaire and physical examination were used to collect qualitative and quantitative primary data on socio-demographic and health characteristics. All information about a child was collected by interviewing the household head. Examination of child eyes was done by trained nurses. Two stage cluster sampling was employed to identify the study subjects. For the purpose of determining the sample size, we estimated the prevalence of trachoma for children aged 1-9 years old to be 37.00% [15]. We have also assumed a design effect of 4.2 (for cluster sampling), a confidence interval of 95% and a maximum allowable error of 10%. These assumptions led to a sample size of 377 children. Out of 17 similarly populated Kebeles (the smallest administrative units in Ethiopia, like a ward or a county in some countries) in the district, six Kebeles were randomly selected. From each selected Kebeles all the children aged 1-9 years old were considered eligible for inclusion in the study. Before the start of the study, Kebele leaders were informed of the study and asked to assist in giving information and consent for examination. Trachoma testing was performed on consenting households.

The study was approved by the zonal health department. All enumerators were trained before initiation of the survey and a pilot test was undertaken in one of the neighboring Kebeles (not included in the final data) to test the procedures and the examiners. Six nurse data collectors and two ophthalmic supervisors with related work experience were recruited. The data collectors and supervisors were trained for two days on how to sample eligible children, administer the questionnaire, eye examination (anthropometric measurements) and address problems in the field. All the training was delivered by the investigator and the two optometrists. All the 377 sampled children were examined for trachoma using the WHO simplified clinical grading system. Interviews and observations were used to assess risk factors. The child's face assessment was carried out before the trachoma examination for face cleanness, discharge and flies on the face. Children's eye examination was done using instrument.

The trachoma grader wearing 2.5x loupes and torch assessed each eye for the active trachoma using the WHO simplified grading scheme: Six trained examiners have to achieve at least 95% inter-observer agreement in identifying active trachoma status. An ordinal severity score of active trachoma comprising three categories was then assigned to all eligible subjects on the bases of the worst-affected eye: 1=*noTF, no TI (no active trachoma); 2=TF only(moderately active trachoma); and 3=TI (severely active trachoma)*. Therefore, the response variable was measured by examining children's eyes using WHO standardized grading system. The risk factors that were assumed to influence or predict the risk outcomes (active trachoma status), were comprised of socio-economic, demographic, hygiene-sanitation and environmental variables that included gender, age, family size, educational level of the mother, monthly income of the family, face washing frequency, use of soap to wash face, where the child spent most of the time, presence of cattle in the living room, whether cooking place is within the living room, access to water source, amount of water fetched per day, fly density, waste/garbage disposal, household crowdedness and latrine availability. Trachoma status, age, gender, frequency of face washing and use of soap to wash face were collected at child level. The remaining variables were all collected at household level.

The gender distribution of children was 196 (52%) female and 181 (48%) male. Reportedly 21.2% of the children spent most of their time at school while 44.6% spent most of their time at home and 34.2% playing on the field. About 17.0% of the children wash their faces at least three times a day. Of the 377 children, 59.0% of them do not use soap to wash their faces. The number of households with pit toilet was 188 (49.8%) while 189(50.2%) households have no (pit) toilet (i.e., use open field). In terms of the waste/garbage disposing, 46.4% the households dispose waste/garbage in a pit. About 79.5% of the households cook in the living room. Likewise the 86.7% of the households kept their cattle within family house.

### Statistical model

Logistic regression is used to predict the probability of the risk outcomes on the basis of the risk factors. Moreover, the effect size of the risk factors on the risk outcomes can be determined from the logistic regression odds ratios. Ordinary logistic regression computes statistics under the assumption that the sample is drawn from a simple random sampling [16]. Survey logistic regression models have the same theory as ordinary logistic regression models. The difference between ordinary and survey logistic is that survey logistic accounts for the complexity of survey designs [17, 18]. But, for data from simple random sampling, the survey logistic regression model and the ordinary logistic regression model are identical. Most sample survey data are collected from a probability-based complex sample design. In order to make statistically valid inferences for the population, the sample design should be incorporated in the data analysis. Thus, survey logistic is developed based on logistic regression with survey data. Unlike the ordinary logistic regression, estimation of the standard errors of the parameter estimates is very complicated for data that come from complex designs. The complexities in variance estimation arise partly from the complicated sample design procedure imposed. Therefore, the incorporation of sampling information is important for the proper assessment of the variance of a statistic. Since specific sample designs are particularly implemented for increasing the efficiency of a statistic, their incorporation in the variance estimation methodology is of major importance [19–21]. Variances of the survey logistic regression parameters and odds ratios are computed by using either the Taylor series (linearization) method or replication (resampling) methods [21–24]. The Taylor linearization method is generally the default in the commonly used software such as SUDAAN, Stata and SAS survey procedures. In particular we apply the ordinal survey logistic regression model to the data, which incorporates the ordinal nature of the dependent variable. The model that incorporates the ordinal nature of the risk outcome is called the cumulative logit model (proportional odds model). Ordinal survey logistic models are extensively used in many studies, and the literature on ordinal logistic modeling is widely available [25–27].

## Results

The data analysis for this study was done using SAS version 9.3 procedure called survey logistic. The Taylor linearization method was used for the variance estimations. However, the replication based-variance estimation and the Taylor linearization with Morel adjustment options were performed and checked if the conclusion changed from that of the linearization method. The results from these two approaches led to the same conclusion. The Akaike information criterion, the negative of twice the log likelihood (-2 logL), and the deviance were used to compare alternative models during model selection. The change in the deviance was used to measure the extent to which the fit of the model improves when additional variables were included. To avoid confounding effects, the model was fitted in two steps. At stage one a model consisting only of the main effects was fitted. In stage two possible combinations of up to 3-way interaction terms were added one at a time and assessed to further avoid and mitigate the problem of confounding. The best fit proportional odds model was identified to be the main effects and five 2-way interactions and two 3-way interactions. We re-evaluated the adequacy of this model fit using a global goodness of fit test. The chi-square score for testing the proportional odds goodness of fit was 50.82, which is not significant with respect to a chi-square distribution with 41 degrees of freedom (p-value=0.1402). This indicates that the proportional odds assumption is reasonable. The predictive concordance power of the model was 91.5%.

Table 1 presents parameter estimates of the final proportional odds model and test of significance. The type 3 effect tests the significance of the risk factor. Among all socio-economic, demographic, hygienic-sanitation and environmental factors, keeping cattle in the house was found to be the significant main effect. On the other hand, monthly family income and where the child spends most of the time; frequency of face washing and use of soap to wash face; where the household disposes the garbage and fly density; where the household disposes the garbage and where the child spends most of the time, and where the child spent most of the time and the availability of pit toilet facility have significant 2-way interaction effects on the level of children trachoma. The two 3-way significant interactions were where the child spends most of the time, fly density and availability of pit toilet and; where the child spent most of the time, fly density and where the household dispose the garbage. The estimates and odds ratios in Table 1 indicate the relative differences among the indicated levels of the factor. The negative estimates reflect the tendency of the cumulative probability starting at the “no active trachoma” end of “severely active trachoma” decreases as factor level changes, and the positive estimates suggest increase at the indicated factor level. For example, estimate of -1.983 and odds ratio of 0.138 in the “cattle in the house” row indicate that the cumulative probability decreases for the “yes” category; and the odds of a child from a household who keeps cattle in the house being in lower trachoma level is 0.138 times the odds of a child from a household who do not keep cattle in house being in the lower trachoma level. In other words, keeping cattle in the house, compared with not keeping cattle in the house, has a multiplicative impact of 0.138 in the estimated odds of trachoma level “no active trachoma” (instead of “moderately active trachoma”, or “severely active trachoma”), and in the estimated odds of trachoma level “moderately active trachoma” (instead of “severely active trachoma”).

**Table 1:** Socio-economic, demographic and environmental effect on trachoma level

Parameter	Estimate	Odds Ratio	P-Value	95% Confidence limits		Type 3 Effect p-value
Intercept 1: No active trachoma	5.277	195.774	0.002	7.016	5462.708	
Intercept 2: Moderate trachoma	8.98	7943.97	<0.0001	710.522	88814.030	
Gender (Ref=Male): Female	0.697	2.007	0.213	0.671	6.005	0.213
Age	-0.006	0.994	0.483	0.978	1.011	0.483
Mother's Education (ref=literate): Illiterate	-0.055	0.947	0.911	0.363	2.473	0.911
Cattle in the house (Ref= No): Yes	-1.983	0.138	0.0003	0.047	0.401	0.0003
Cooking in the house (Ref=No): Yes	-0.119	0.888	0.791	0.370	2.132	0.791
**Children spent time most the time (Ref=School)**						0.534
Home	-5.908	0.003	<.0001	0.000	0.018	
Field	-4.844	0.008	0.014	0.000	0.376	
Face washing frequency per day (Ref=once)						<.0001
Three or more	1.589	4.899	0.058	0.945	25.391	
Twice	1.467	4.337	0.004	1.593	11.805	
Use of soap to wash face (Ref=No):Yes	0.614	1.847	0.002	6.341	1.002	<0.002
Distance to fetch water	0	1	0.052	2.718	1.053	0.052
Number of people sharing one room(Ref= <5): >5	0.789	2.201	0.113	9.034	1.120	0.069
**Fly density(Ref=zero)**						<.0001
More than four	-9.516	0	<.0001	0.000	0.000	
One to four	-11.699	0	<.0001	0.000	0.000	
Garbage dispose (Ref= Open field):Pit	-18.92	0	<.0001	0.000	0.000	0.003
Toilet facility (Ref= Open field):Pit	18.132	>1000	<.0001	>1000	>10000	<.0001
Monthly family income	0.027	1.027	<.0001	1.014	1.040	<0.001
**Monthly family income and where the child spend most of the time (Ref=School)**						<0.0001
in home	-0.015	0.985	0	0.977	0.993	
field	-0.017	0.983	<.0001	0.975	0.991	
**Face washing frequency and Use soap (Ref=once and No)**						0.0069
Three or more and Yes	2.457	11.664	0.455	0.019	7313.666	
Twice and Yes	2.663	14.341	0.003	2.422	84.919	
**Child spend most of the time and Toilet facility (Ref=School and Open field)**						<0.0001
Home and Pit	-18.328	0	<.0001	0.0000	0.000	
Field and Pit	-19.635	0	<.0001	0.0000	0.000	
**Fly density and Garbage dispose (Ref=zero and Open field)**						<0.0001

**Figure 1:**
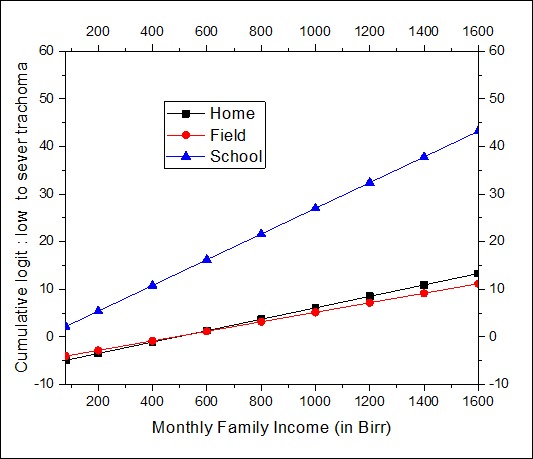
The effect of monthly family income by where the child spend most of the time

**Figure 2:**
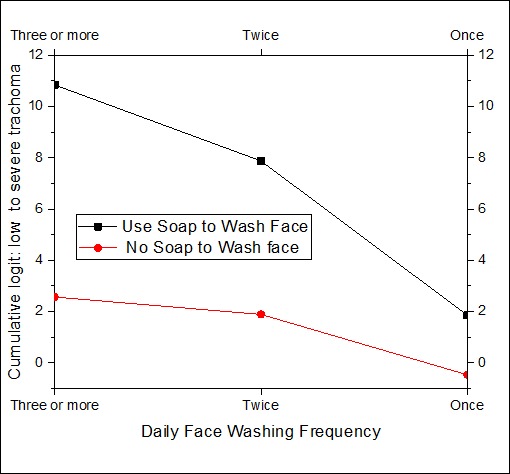
Joint effect of daily face washing frequency and use of soap to wash face

**Figure 3:**
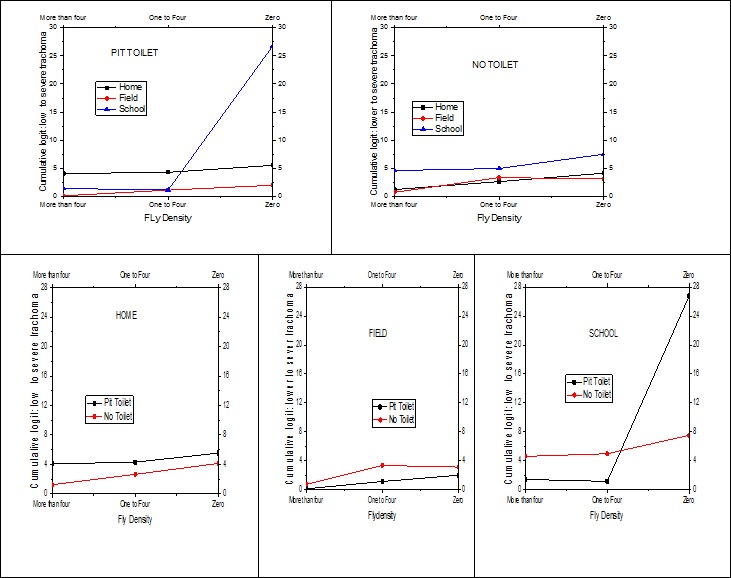
The 3-way effect of toilet facility, where the child spends and fly-density

Interpretation of the effect of the risk factor involved in interactions should be performed with special care. Graphical displays are helpful to assess the effect of one risk factor at a given value of the other interaction term. The significant 2-way interactions which are also in the 3-way interaction should be interpreted in the higher order of interaction. Figure 1 displays the distribution of the cumulative logit of trachoma against monthly family income for places where the child spends most of the time. The minimum and maximum monthly family incomes were 80 and 1600 Birr (Ethiopian Currency) respectively. The estimated cumulative logits given in Figure 1 are within this observed income range. The result indicates that the odds of a child having lower trachoma level increases with his/her family income. However, the rate of increase for the cumulative odds of low level trachoma depends on where the child spends most of his/her time. Evidently there is no difference between playing in the field and at home. The highest rate of increase on the odds of lower level of trachoma is observed for children spending most of their time in school. The odds difference between school, and home and field gets greater as the family income increases. Figure 2 displays the joint effect of the number of times the child washes his/her face and the application of soap in washing the face. As the frequency of washing face decreases the odds of having low trachoma level decreases. This is true for both soap users and non-users in the wash. The effectiveness of the soap becomes more evident for children who wash their faces more than one time per day. Figure 3 displays the 3-way effect of where the child spends most of the time, fly density and toilet facility. For children from the pit toilet user households, if the child spends most of the time at home or in the field the cumulative odds of having low level of trachoma instead of severe/moderate level of trachoma is identical for different levels of fly-density on the child's face. On other hand, for children from no toilet household and who spend most of the time at home or playing in the field, as the fly-density on the child's decreases the cumulative odds of having low trachoma instead of moderate/severe trachoma. For the household with pit toilet, irrespective of fly-density, children spending most of the time at home have higher cumulative odds of low trachoma than that of children spending most of the time in the field. But for the household with no toilet, the difference between spending most of the time at home and playing in the field is insignificant. For households who do not have toilet, children who spent most their time at school have higher cumulative odds of low trachoma, irrespective of the density, than that of children who spend most of their time at home or in the field. However, for children from the household with pit toilet, schooling will have very high cumulative odds of low trachoma if only the fly density is zero.

**Figure 4:**
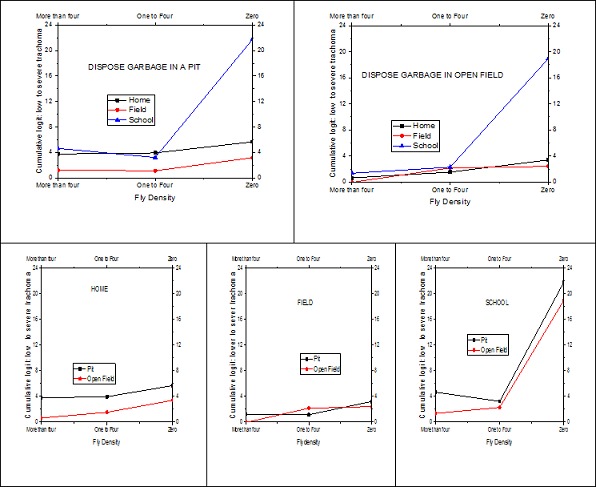
The 3-way effect of garbage disposal, where the child spends and fly-density

Likewise Figure 4 displays the effect of where the child spends most of the time and flies density for households who dispose garbage in a pit and households who dispose garbage in open field. For children from a household who disposes garbage in a pit and if the child spends most of the time at home or playing field the cumulative odds of having low level of trachoma instead of severe/moderate level of trachoma is identical for different levels of fly-density on the child's face. Regardless of the fly density, for garbage disposing pit using household children who spent most their time at home the cumulative odds of having low trachoma is higher than that of otherwise identical children who spend most of their time in the field. On the other hand, for children from a household who disposes garbage in open field if the child spends most of the time at home or in the field, as the fly-density on the child's face decreases, the cumulative odds of having low trachoma instead of moderate/severe trachoma increases in general and the differences between home and field is insignificant. The cumulative odds of low trachoma level increases alarmingly for children who spent most of their time in school for both households who dispose their garbage in the pit or in the open field when the fly density is zero.

## Discussion

In this study the determinants of the risk factors of active trachoma in rural areas of Ethiopia were investigated using ordinal survey logistic regression. Keeping cattle in the same room as people overnight was found to be a significant protective factor. This protective effect may simply be due to a correlation between cattle ownership and wealth, which is in turn associated with better outcomes concerning trachoma. Bear in mind that the interaction effect of cattle keeping in the house and monthly income was not significant in this study (p=0.3467). However, other studies in Ethiopia [6, 28], in South Sudan [8], and in Tanzania [29] showed otherwise. Their argument was that flies may breed on animal faeces which in turn increase exposure of children to flies. However, another study from Ethiopia [30] confirmed that neither cattle ownership nor the presence of cattle in the village has a major role in the size of the fly population; instead, the major determinant seems to be the way in which the cattle were kept. Thus, one of the future directions of this study will be to collect information on how the cattle were housed together with the family. Children from a family getting more income are more likely to have low level of active trachoma as compared to families having less monthly income. This may be because children from a relatively well to do family will have access to sanitary materials and a better information. Similar finding was reported in Ethiopia [3]. The result of our study showed that the effect of income will be prominent only if the child attends school. Facial hygiene has a positive effect on the reduction of the risk of trachoma. The effect of facial water, sanitation and hygiene (WASH) is enhanced by the daily frequency and application of soap in the WASH. The odds of low trachoma for children who wash their faces with water and soap once a day is equal to that of otherwise identical children who wash their faces three or more times with water only (Figure 2). Therefore it is advisable to wash children faces with water and soap at least once a day.

One of the most important interaction factors associated with low level of active trachoma was the use of pit toilet, where the child spends most of time and fly-density. This is a reasonable finding as using pit latrines may decrease the possibility of fly breeding in the area and hence the fly density. This association between low level of active trachoma and access to a latrine is consistent with previous studies [6, 11, 31]. However some other trachoma risk factor studies have reported that risk of trachoma was not associated with the use of pit latrine [28, 32]. Such controversy arises due to unawareness of the interaction effect. For instance, if one compares children who spent most of their time at school but have no household toilet and children who spent most of their time at home but have household toilet, the difference in the cumulative odds of trachoma is insignificant regardless of the fly-density (Figure 3). Or if one compares the cumulative odds of pit-toilet and no pit- toilet household children who spent most of their time playing in the field the difference is insignificant irrespective of the fly-density. Accordingly one may conclude that toilet facility has no association with the risk of trachoma outcomes [28, 32]. Thus familiarity with interaction effects is essential in such a risk factor assessment. Similarly, holistic interpretation of the joint effect of disposing garbage in a pit, where the child spends most of the time and fly-density may help to resolve the inconsistencies and controversies in similar studies. The use of pit instead of open field to dispose garbage is found to be significantly associated with low level of active trachoma in a study in Niger [31], but not supported in a community-based trachoma survey in the northern part of Ethiopia [33]. Again this anomaly might be lack of knowledge in the interaction effects. If one considers children who spent most of their time in the field (Figure 4), the conclusion is in line with the study in Niger [31] but if one considers children who spent most of the time at home the result is in line with another study in Ethiopia [33].

## Conclusion

This article was aimed to make a contribution to the study of trachoma risk factors by adopting a model which is flexible enough to account for the complexity of the survey and to include the interaction effects. The model results contributed to clarify some of the possible inconsistencies in similar survey results. The list of factors that we have used in the study is by no means exhaustive, but accommodated almost all the factors considered in similar studies. The proposed model capability and novelty is, however, well demonstrated by this survey data. The inclusion of interaction terms to the model greatly expands our understanding of the relationships among the risk factors in the model. The presence of a significant interaction indicates that the effect of one risk factor on the risk outcome is different at different values of the other risk factor. Accordingly the presence of significant interaction term drastically changes the interpretation of the main effects. As demonstrated in this study, careful plotting of the results is a crucial aspect of explaining the substantive implication of the interaction. Likewise, the recommendations and interventions which may be implied from such study should not be in isolation from one another. For example, improving the household income level in order to reduce the risk of children trachoma seems commendable the impact would be minimal unless the household's children have access to school.

### What is known about this topic

- Several socioeconomic and lifestyle factors were found to independently affect the risk of trachoma (interactions between risk factors were not clearly indicated).
- The methods of analyses used do not take into account the complexity of survey techniques (the methods used assume simple random sampling even though in practice complex surveys were taken).

### What this study adds

- In this study two stage cluster sampling was used to collect data and survey logistic regression model, which accounts for the complexity of survey designs and interaction effects, was used.
- Two and three way interaction effects were found significant. The inclusion of interaction terms to the model greatly expands our understanding of the relationships among the risk factors in the model and contributed to clarify some of the possible inconsistencies in similar survey results.
- The Taylor linearization method was used for the variance estimations. However, the replication based–variance estimation and the Taylor linearization with Morel adjustment options were performed and checked if the conclusion changed from that of the linearization method.

## References

[1] Hu VH, Harding-Esch EM, Burton MJ, Bailey RL, Kadimpeul J, Mabey DCW (2010). Epidemiology and control of trachoma: systematic review. Tropical Medicine and International Health..

[2] Frick KD, Hanson CL, Jacobson AA (2003). Global burden of Trachoma and economics of the disease. Am J Trop Med Hyg..

[3] Ketema K, Tiruneh M, Woldeyohannes D, Muluye D (2012). Active trachoma and associated risk factors among children in Baso Liben District of East Gojjam, Ethiopia. BMC Public Health..

[4] Burton MJ, Mabey DCW (2009). The Global Burden of Trachoma: A Review. PLoS Negl Trop Dis..

[5] Mariotti SP, Pascolini D, Rose-Nussbaumer J (2009). Trachoma: global magnitude of a preventable cause of blindness. Br J Ophthalmol..

[6] Mpyet C, Goyol M, Ogoshi C (2010). Personal and environmental risk factors for active trachoma in children in Yobe state, north-eastern Nigeria. Tropical Medicine and International Health..

[7] Baggaley RF, Solomon AW, Kuper H, Polack S, Massae PA, Kelly J, Safari S, Alexander NDE, Courtright P, Foster A, Mabey DC (2006). Distance to water source and altitude in relation to active trachoma in Rombo district, Tanzania. Tropical Medicine and International Health..

[8] Ngondi J, Matthews F, Reacher M, Onsarigo A, Matende I, Baba S, Brayne C, Zingeser J, Emerson P (2007). Prevalence of Risk Factors and Severity of Active Trachoma in Southern Sudan: An Ordinal Analysis. Am J Trop Med Hyg..

[9] Berhane Y (2007). Prevalence of trachoma in Ethiopia. Ethiopian Journal of Health Development..

[10] Edwards T (2008). Risk factors for active trachoma and Chlamydia trachomatis infection in rural Ethiopia after mass treatment with azithromycin. Tropical Medicine and International Health..

[11] Golovaty I (2009). Access to Water Source, Latrine Facilities and Other Risk Factors of Active Trachoma in Ankober, Ethiopia. PLoS ONE..

[12] Haileselassie T, Bayu S (2007). Altitude-a risk factor for active trachoma in Southern Ethiopia. Ethiopian Medical Journal..

[13] Ngondi J (2008). Risk factors for active trachoma in children and trichiasis in adults: a household survey in Amhara Regional State, Ethiopia. Transactions of the Royal Society of Tropical Medicine and Hygiene..

[14] Alemayehu W, Melese M, Geyid A, Mekonnen Y, Tilahun D, Asfaw T (2007). Seasonal and altitudinal variations in fly density and their association with the occurrence of trachoma, in the Gurage zone of central Ethiopia. Ann Trop Med Parasitol..

[15] Vinke C, Lonergan S (2011). Social and environmental risk factors for trachoma, a mixed method Kembata zone of SNNP. Canadian Journal of Development Studies..

[16] McCullagh P, Nelder J (1989). Generalized Linear Models, seconded.

[17] Morel JG (1989). Logistic Regression under Complex Survey Designs. Survey Methodology..

[18] Lehtonen R, Pahkinen E (1995). Practical Methods for Design and Analysis of Complex Surveys.

[19] Lohr SL (1999). Sampling: Design and Analysis.

[20] Korn E, Graubard B (1999). Analysis of Health Survey.

[21] SAS (2008). SAS 9.2: SAS/STAT^®^ 9.2 User's Guide The SURVEYLOGISTIC Procedure (Book Excerpt).

[22] Rao JNK, Wu CFJ, Yue K (1992). Some Recent Work on Resampling Methods for Complex Surveys“. Survey Methodology..

[23] Särndal CE, Swensson B, Wretman J (1992). Model Assisted Survey Sampling.

[24] Lee ES, Forthofer RN (2006). Analyzing Complex Survey Data.

[25] Habyarimana F, Zewotir T, Ramroop S (2014). A proportional odds model with complex sampling design to identify the determinants of malnutrition of children under five years in Rwanda. Mediterranean Journal of Social Sciences..

[26] Gameroff MJ (2005). Using the proportional odds model for health-related outcomes: Why, When, and How with various SAS procedure, proceedings of the thirtieth annual SAS users group international conference.

[27] Agresti A (2010). Categorical Data Analysis.

[28] Cumberland P, Hailu G, Todd J (2005). Active trachoma in children aged three to nine years in rural communities in Ethiopia: prevalence, indicators and risk factors. Trans R Soc Trop Med Hyg..

[29] Polack S (2006). The relationship between prevalence of activetrachoma, water availability and its use in a Tanzanian village. Transactions of the Royal Society of Tropical Medicine and Hygiene..

[30] DE Sole G (1987). Impact of cattle on the prevalence and severity of trachoma. British Journal of Ophthalmology..

[31] Abdou A, Nassirou B, Kadri B (2007). Prevalence and risk factors for trachoma and ocular Chlamydia trachomatis infection in Niger. British Journal of Ophthalmology..

[32] Ngondi J, Gebre T, Shargie EB (2008). Risk factors for active trachoma in children and trichiasis in adults: a household survey in Amhara Regional State, Ethiopia. Transactions of the Royal Society of Tropical Medicine and Hygiene..

[33] Mesfin MM, de la Camera J, Tareke IG, Amanual G, Araya T (2006). A community-based trachoma survey: prevalence and risk factors in the Tigray region of northern Ethiopia. Ophthalmic Epidemiol..

